# Supplementary material for: Body Composition Profiles of Applicants to a Physical Education and Sports Major in Southeastern Mexico
Source: Int J Environ Res Public Health. 2022 Nov 25;19(23):15685. doi: 10.3390/ijerph192315685 (PMC9735584; doi:10.3390/ijerph192315685)
Supplement: Supplementary file 1 [file ijerph-19-15685-s001.zip › ijerph-2028820-supplementary.pdf]

Table S1. Means, standard deviations, and correlations with confidence intervals

Variable	Mean	SD	1	2	3	4	5	6	7	8
1. Age	18.09	0.28								
2. Stature	1.67	0.09	.06<br>[-.05, .17]							
3. BM	66.13	12.40	.08<br>[-.03, .18]	.59**<br>[.51, .65]						
4. IMC	23.48	3.53	.05<br>[-.05, .16]	.06<br>[-.05, .17]	.84**<br>[.80, .87]					
5. FM	23.71	9.86	-.02<br>[-.13, .09]	-.39**<br>[-.48, -.29]	.24**<br>[.13, .34]	.56**<br>[.48, .63]				
6. MM	37.01	7.50	.04<br>[-.07, .15]	.50**<br>[.42, .58]	.01<br>[-.10, .11]	-.32**<br>[-.41, -.22]	-.81**<br>[-.84, -.77]			
7. MA	28.10	12.03	.08<br>[-.03, .19]	.17**<br>[.06, .27]	.81**<br>[.77, .85]	.87**<br>[.84, .90]	.57**<br>[.49, .64]	-.41**<br>[-.50, -.32]		
8. BMR	1550.17	227.40	.07<br>[-.04, .18]	.67**<br>[.61, .73]	.87**<br>[.84, .89]	.61**<br>[.54, .68]	-.16**<br>[-.26, -.05]	.43**<br>[.33, .51]	.55**<br>[.47, .62]	
9. VFL	5.24	2.80	.03<br>[-.08, .14]	.16**<br>[.05, .27]	.81**<br>[.77, .84]	.88**<br>[.85, .90]	.35**<br>[.25, .44]	-.12*<br>[-.23, -.01]	.77**<br>[.72, .81]	.73**<br>[.67, .77]

Data are expressed as mean and standard deviation. Values in square brackets indicate the 95% confidence interval for each correlation. BM = body mass; BMI = body mass index; %FM = percentage of fat mass; %MM = percentage of muscle mass; MA = metabolic age; BMR = basal metabolic rate; VFL = visceral fat level; * P < 0.05; ** P < 0.01.
